# Development of a Cissus quadrangularis-Doped Extracellular Matrix and a Hyaluronic Acid-Incorporated Scaffold for Periodontal Regeneration: An In Vitro Study

**DOI:** 10.7759/cureus.56507

**Published:** 2024-03-19

**Authors:** Balaji Ganesh S, Abraham Sabu, G Kaarthikeyan, Rajalakshmanan Eswaramoorthy, Priyangha P T

**Affiliations:** 1 Periodontics, Saveetha Dental College and Hospitals, Saveetha Institute of Medical and Technical Sciences, Saveetha University, Chennai, IND; 2 Dentistry, Saveetha Dental College and Hospitals, Saveetha Institute of Medical and Technical Sciences, Saveetha University, Chennai, IND; 3 Biomaterials, Saveetha Dental College and Hospitals, Saveetha Institute of Medical and Technical Sciences, Saveetha University, Chennai, IND

**Keywords:** biomedical, tenogenesis, scaffold, hydrogel, extracellular matrix, hyaluronic acid, dental

## Abstract

Purpose: The study aimed to analyze whether adding *Cissus quadrangularis* (CQ) extract and the extracellular matrix of ovine tendon (TENDON) increases the regenerative potential of mesenchymal stem cells produced in hyaluronic acid (HA) scaffolds for tenogenesis.

Materials and methods: Fifty grams of powdered CQ was mixed with 250 mL of ethanol to prepare the extract. Two grams of hyaluronic acid powder was added to 100 mL of distilled water to make the HA solution. The ovine tendon was decellularized using a mixture of 10% phosphate-buffered saline (PBS), sodium dodecyl sulfate (SDS), and Triton-X. The hydrogel samples were prepared by mixing the extracellular matrix of tendon, HA, and CQ, after which they were divided into study groups such as HA, HA + CQ, HA + TENDON, and HA + CQ + TENDON. Scanning electron microscopy (SEM) analysis, swelling analysis, differentiation analysis, compression test, compatibility assay, and tenogenesis assay were later conducted.

Results: The morphology of the samples was analyzed using SEM. Low levels of swelling of the hydrogels were observed. Cells were found to be viable and showed good differentiation and tenogenesis. Optimal compression levels were observed, and the properties of the prepared hydrogels were satisfactory.

Conclusion: The results suggest that the addition of CQ considerably increases the tenogenic potential of the extracellular matrix/HA scaffold. Hence, it can be used as a regenerative material for periodontal tissue regeneration.

## Introduction

Tendon is defined as fibrous tissues that are densely packed with collagen fiber bundles, and its predominant function is to provide muscle-to-bone connection [1]. They are considerably stronger than muscles and can provide resistance against compressive and tensile forces. Tenocytes are responsible for synthesizing collagen (predominantly type I) and other parts of the ECM. The periodontal ligament is comparable to a tendon/ligament to bone insertion. Even after years of research, they remain a persistent clinical challenge. The Global Burden of Disease 2016 has detailed that the incidence and prevalence of musculoskeletal disorders accounted for around 1.27 billion and 652 million, respectively [2]. Tendon injuries are caused by intrinsic factors and extrinsic factors together or independently. Current treatment methods cannot restore the structural, functional, and biochemical properties of repaired tendon tissue compared to native tissue. Treatment modalities for tendon injury include steroid injections, low-intensity pulsed ultrasound shockwaves, and physiotherapy. The main demerit of these treatment methods is the long duration they require, apart from the fact that they may only reduce pain. Surgical treatment modalities are required in acute tendon injury. Autografts, allografts, xenografts, and prosthetic devices are the treatment of choice in cases of severe tendon injuries [3]. Still, there are certain disadvantages, such as donor site morbidity and risk of disease transmission, and they are limited by long-term function. Hence, alternate methods of treatment are getting more attention.

*Cissus quadrangularis *(CQ) is also commonly known as veldt grape and devil's backbone. It belongs to the family of Vitaceae (grape family). It can reach a height of 1.5 m (4.9 ft) and possesses quadrangular-sectioned branches with internodes 8-10 cm (3-4 in) long and 1.2-1.5 cm (0.5-0.6 in) wide. These species are native to tropical Asia and Arabia. They are widely used in Ayurvedic medicine to treat injured ligaments and broken bones. Recent studies on CQ have confirmed the presence of anti-osteoporotic, anti-ulcer, antioxidant, anticancer, anti-analgesic activity [4], and antibacterial activity [5]. It comprises 90% of collagen, mostly type I [6]. Hyaluronic acid (HA), a natural linear polysaccharide, has a molecular weight ranging from 1,000 to 10,000,000 Da. It has interglycosidic linkage and alternating disaccharide units of D-glucuronic acid and N-acetyl-D-glucosamine with beta(1 → 4) linkage. It is also known to be biocompatible and biodegradable. It is present abundantly in extracellular matrices and synovial fluid. It plays a prominent role in wound healing, accelerating cell differentiation and motility during development. In tissue engineering, it is used in the fabrication of hydrogels. It is used as a cell-seeding base for the scaffold. It is a bioactive compound that provides rigidity and viscoelasticity and helps improve tenogenesis [7]. It is one of the fundamental components present in tendon tissue. HA injection is used to treat osteoarthritis. It also has anti-inflammatory properties and enhances the cellular activity of fibroblasts and their adhesivity. It also helps to improve extracellular matrix (ECM) synthesis and proliferation.

Mesenchymal stem cells are widely used for tissue engineering and ethnogenesis. They are commonly found in tendons, bone marrow, and adipose tissue. They can accelerate and improve the quality of tendon healing by enhancing the tenogenic properties of tendon resident cells. In this study, mesenchymal stem cells were used instead of embryonic stem cells to prevent the risk of teratoma formation [8]. The hydroxyapatite scaffold with stem cells incorporated has proved to be effective for periodontal regeneration [9].

Previously, studies have been conducted with human adipose-derived stem cells, and rabbit-derived stem cells seeded on decellularized tendon/ligament ECM showed proliferation and differentiation [10, 11]. Another study was conducted where bovine Achilles tendon ECM was incorporated with human adipose-derived stem cells, which showed viability and proliferation [12]. Yet another study has proved the efficacy of bovine-derived hydroxyapatite combined with CQ in the treatment of intrabony defects has yielded favorable results [13, 14]. The pharmacological effects of the plant have also been proved, which substantiates its application in periodontal regeneration [15]. Thus, this study aimed to analyze whether adding a CQ extract and extracellular matrix of ovine tendon increases the regenerative potential of mesenchymal stem cells produced in HA scaffolds for tenogenesis.

## Materials and methods

Sample preparation

Leaves of CQ were collected from an herbal garden and dried. After drying, the leaves were ground into a fine powder using a mortar and pestle. Extraction of secondary metabolites from CQ was done, for which 50 g of powdered CQ was added to 250 mL of ethanol and placed in a shaker for 24 hours at 120 rpm. The solution was left to settle for 24 hours, and the supernatant was collected and subjected to rotary evaporation to obtain the CQ extract. Methacrylate HA fabrication was the next step, for which 2 g of HA powder was added to 100 mL of water and stirred until a homogeneous viscous HA solution was obtained [16]. Finally, the ovine tendon (TENDON) was removed from the carcass and reduced into minute fragments, immersed in 20 mL of 10% phosphate-buffered saline (PBS). One gram of sodium dodecyl sulfate (SDS) followed by 200 µL of Triton-X and 100 mL of distilled water prepared the decellularization fluid. Then, 25 mL of the decellularization fluid was added to the tendon sample and placed in a shaker at 37 °C until foam formed. The froth was removed from the decellularization fluid until there was no froth formation. The hydrogel samples were prepared by mixing the extracellular matrix of the tendon, HA, and the natural product CQ as follows and solidifying them in sample wells. They were divided into four groups: HA, HA+TENDON, HA+CQ, and HA+TENDON+CQ.

Scanning electron microscopy (SEM) analysis

The hydrogel samples were fixed in a 4% paraformaldehyde solution overnight and dehydrated through successive graded ethanol baths (10-100%). Later, the dehydrated samples were fixed on aluminum stubs and coated with gold via a sputter coater at 37 °C. Then, the morphological characteristics of the samples were visualized using a scanning electron microscope (JEOL JSM-IT800 FE-SEM, JEOL Ltd, Tokyo, Japan).

Swelling analysis

Some of the hydrogel samples were dry weighed, immersed in 5 mL of 10% PBS solution at 37 °C for an hour, and then weighed to test the swelling and fluid absorption level. The hydrogel was weighed after being blotted with tissue paper to remove the excess PBS solution. The swelling ratios were calculated using the following equation: Swelling ratio (SR) = ((W - W0)/W0) × 100% (W is the initial wet weight, and W0 is the initial dry weight).

Compression test

The mechanical strength was tested by subjecting the samples to compressional forces. Using a universal testing machine, the samples were tested for the force required to cause a fracture (Instron ElectroPuls E3000, Instron Corp., Norwood, Massachusetts). The compressive force was applied at a cross-head speed of 1 mm/minute until fracture. A piece of tin foil with a thickness of 0.1 mm was placed between the loading piston and the hydrogel sample. The hemispherical steel head indenter with a diameter of 5 mm was placed on the surface of the hydrogel sample. The value of the maximum force necessary to cause a fracture was recorded in newtons (N), and the compressive stress was recorded in megapascals (MPa).

Differentiation analysis

The hydrogel scaffolds were placed in 24-well plates coated with 0.3% poly(2-hydroxyethyl methacrylate) (polyHEMA) to ensure the cells did not adhere to the polystyrene. The scaffolds were incubated at 37 °C and 5% CO_2_ for seven days. Two milliliters of a conditioned cellular induction medium consisting of Dulbecco's modified Eagle's medium (DMEM) (12-604F DMEM, Lonza AG, Basel, Switzerland) supplemented with 10% fetal bovine serum (FBS) (HiMedia RM9955 FBS, HiMedia Labs Ltd., Chennai, India), and 1% penicillin-streptomycin (PenStrep) was added to each well. The differentiation rates were visualized regularly using a biological microscope (Labomed Microscope, Labomed Inc., Los Angeles, California).

Compatibility assay

The cell viability and proliferation levels were tested by the 3-(4, 5-dimethylthiazol-2-yl)-2, 5-diphenyl-2H-tetrazolium bromide (MTT assay). The samples prepared were seeded in a 96-well ELISA plate with 100 µL per well. Ten microliters of the MTT solution were added to the wells and incubated for three hours at 37 °C. One hundred microliters of dimethyl sulfoxide (DMSO) were added to each well to dissolve the crystalline formazan product formed. After formation, the absorbance was recorded at 570 nm.

Tenogenesis assay (Sirius red staining)

After cellular induction for seven days, the differentiation rates were analyzed microscopically. Then, the induction medium was removed, and the cells were washed with PBS solution. The cells were fixed with 70% ethanol for 30 minutes and washed thrice. The deposited collagen was stained with 0.1% Sirius red (in a saturated solution of picric acid) (Picrosirius Red, Sigma-Aldrich, St. Louis, Missouri) in a saturated aqueous solution of picric acid. To quantify the stained nodules, the stain was solubilized with 0.5 mL of a 1:1 (vol/vol) solution of 0.1% NaOH and absolute methanol for 30 minutes at room temperature. 0.1 mL of the solubilized stain was transferred to the wells of a 96-well plate, and the absorbance was measured at 540 nm. Data were presented as mean ± standard deviation, where n = 3.

## Results

SEM analysis

**Figure 1:**
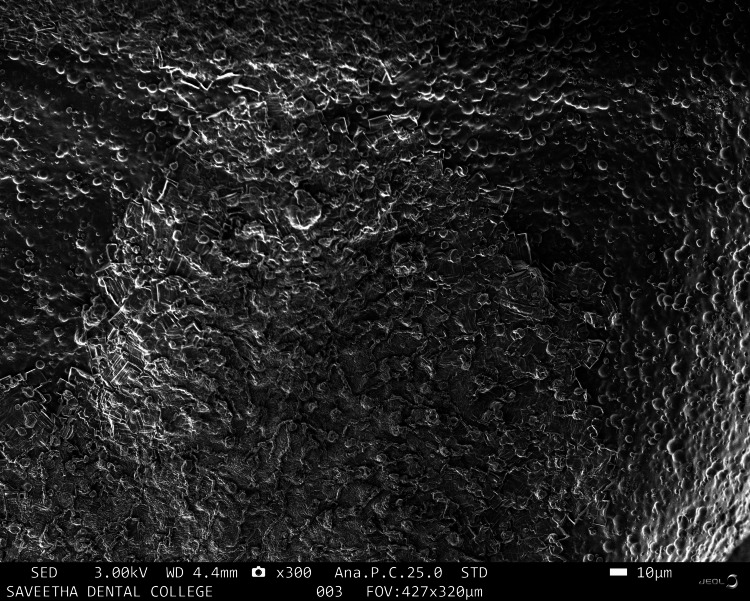
Scanning electron microscopy image of hyaluronic acid

**Figure 2:**
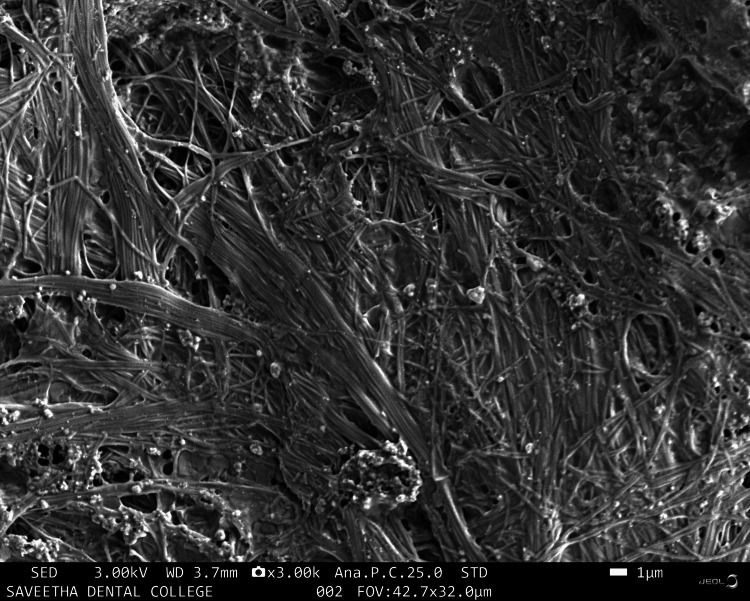
Scanning electron microscopy image of TENDON

**Figure 3:**
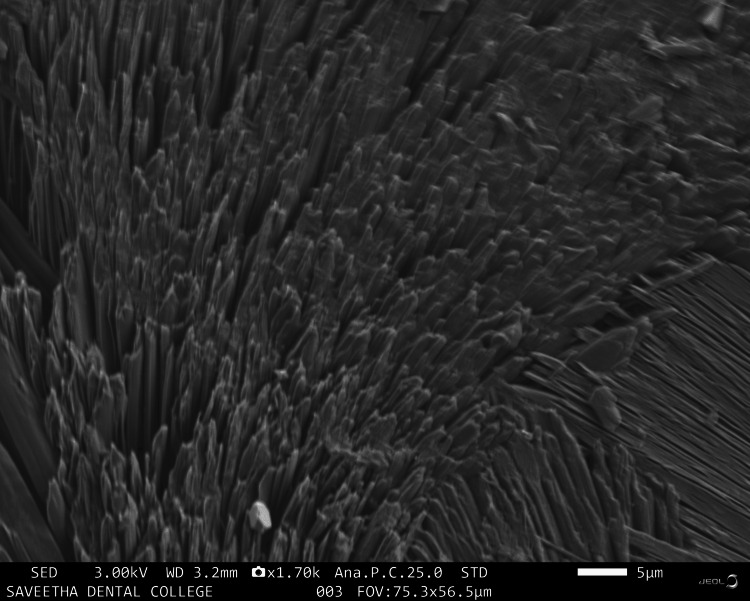
Scanning electron microscopy image of hyaluronic acid +TENDON

**Figure 4:**
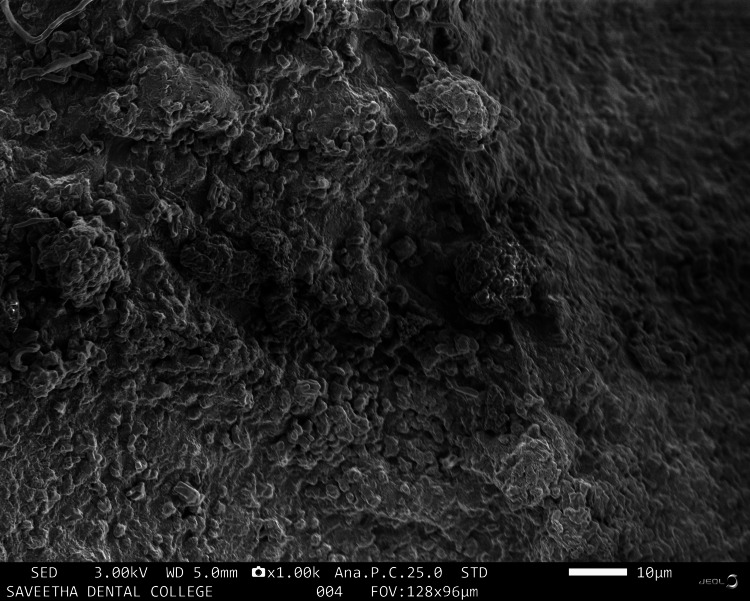
Scanning electron microscopy image of hyaluronic acid + Cissus quadrangularis

**Figure 5:**
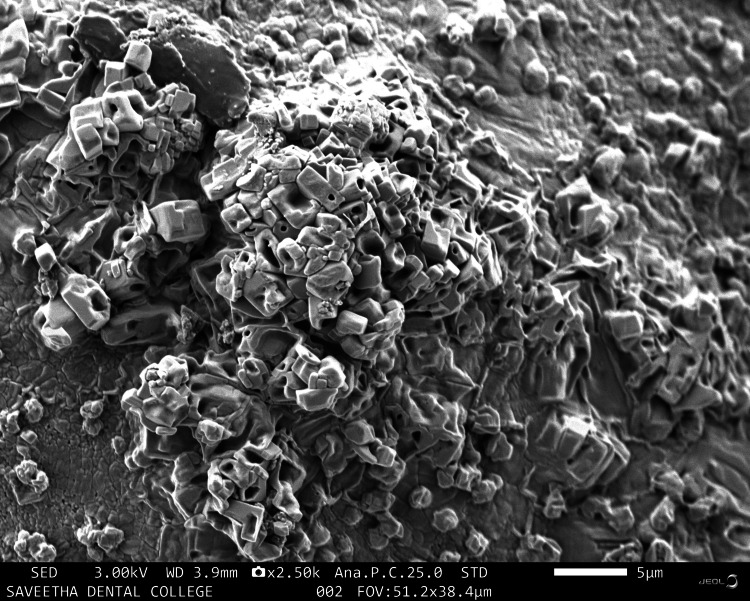
Scanning electron microscopy image of hyaluronic acid + Cissus quadrangularis + TENDON

The characteristics of the molecular morphology of the hydrogel samples were visualized using SEM (JEOL JSM-IT800 FE-SEM, Tokyo, Japan) at 3.00 kV, as described in Figures 1-5. Figure 1 shows irregular and rough morphology with cresting and troughing on the topographical region of the HA sample.

Figure 2 shows fibrous morphology with a very high density of interconnected fibers of the TENDON.

Figure 3 shows collagen fibers bundled over the HA particles with a regular morphology.

Figure 4 shows the microbead-like appearance of the HA+CQ sample.

Figure 5 shows the combined morphology of the prepared HA+CQ+TENDON sample.

Swelling analysis

**Figure 6:**
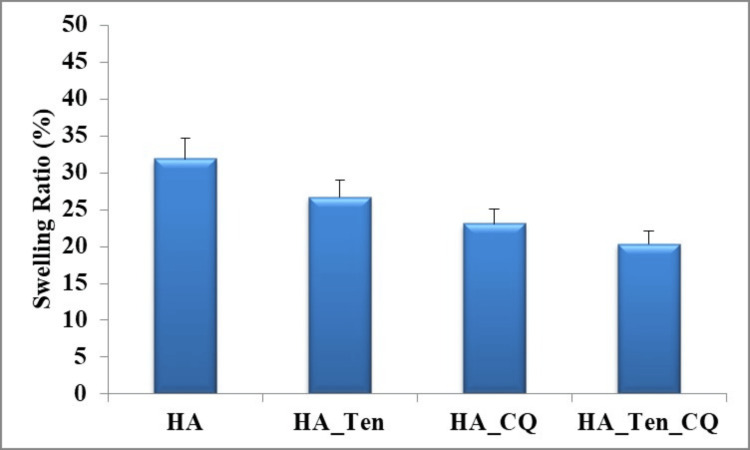
Swelling analysis. The standard deviation in each case is 0.05 (HA), 0.05 (HA_Ten), 0.04* (HA_CQ), and 0.04* (HA_Ten_CQ) * p-value less than 0.05, which is considered statistically significant. HA: hyaluronic acid; HA_Ten: hyaluronic acid + TENDON; HA_CQ: hyaluronic acid +Cissus quadrangularis; HA_Ten_CQ hyaluronic acid + TENDON + Cissus quadrangularis.

Swelling analysis shows the amount of fluid imbibed by each sample. The ratios were calculated for each sample. Figure 6 shows the swelling ratios of HA, HA + TENDON, HA+CQ, HA+CQ+TENDON. The swelling ratio was lowest in HA + TENDON + CQ compared to HA and HA + TENDON so they absorb the least amount of fluid. So the doping of *Cissus Quadrangularis* reduces the absorption capacity of the hydrogel scaffolds.

Differentiation analysis

**Figure 7:**
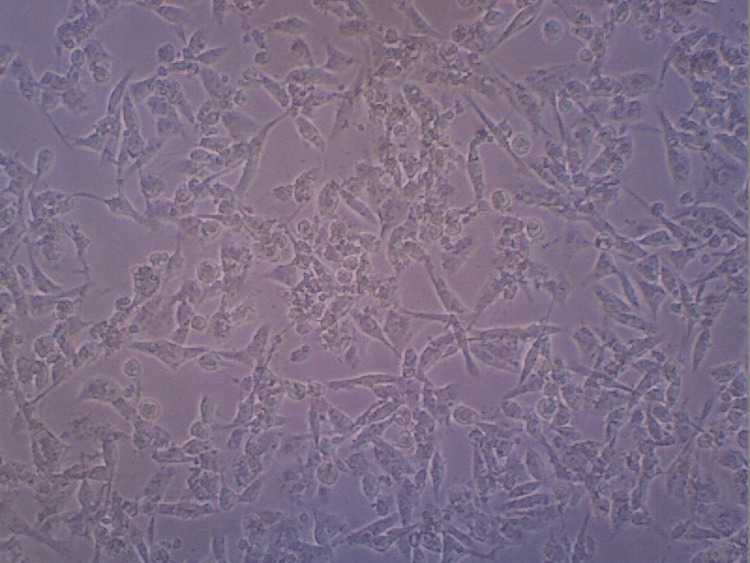
Contrast microscopy image of hyaluronic acid scaffold sample

**Figure 8:**
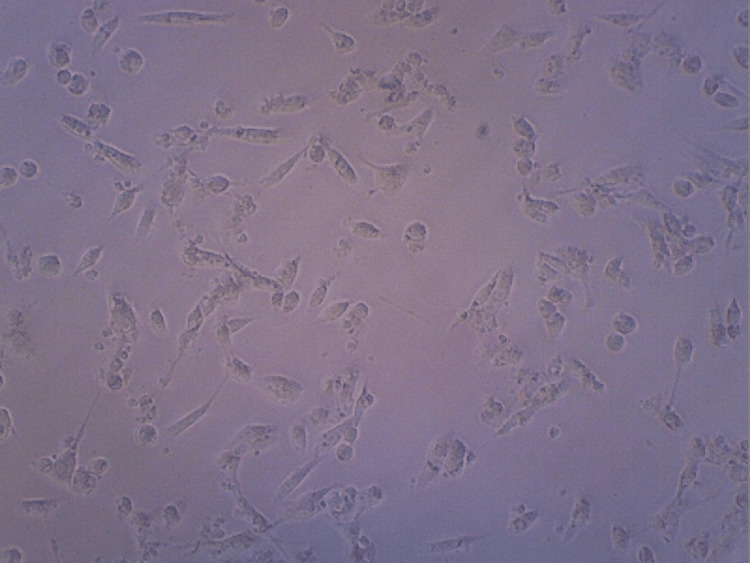
Contrast microscopy image of hyaluronic acid + Cissus quadrangularis

**Figure 9:**
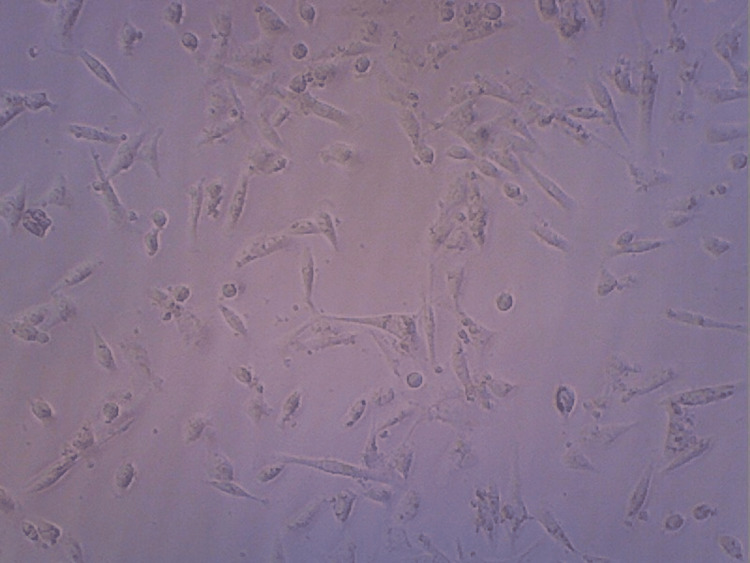
Contrast microscopy image of hyaluronic acid+TENDON

**Figure 10:**
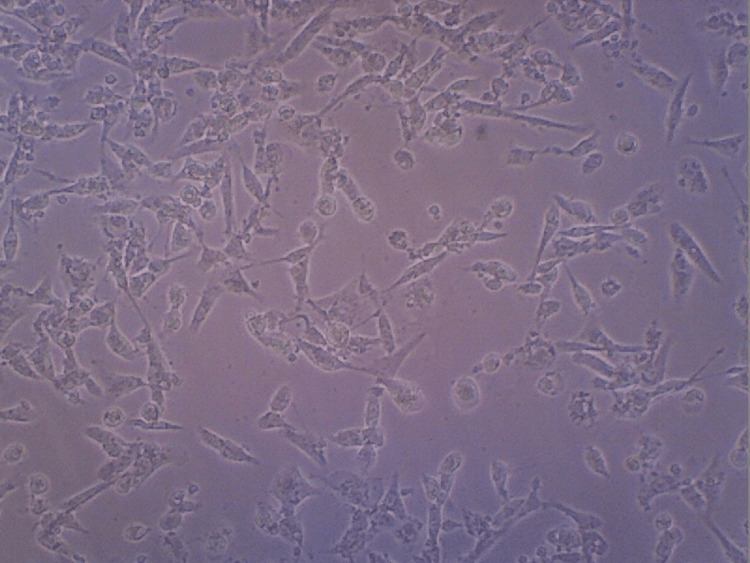
Contrast microscopy image of hyaluronic acid+TENDON+Cissus quadrangularis

Differentiation analysis shows the formation of tendon tissue from the stem cells by visualizing them at 20x in a contrast microscope, as shown in Figures 7-10. Maximum differentiation was observed in both HA and HA + CQ + TENDON groups. Figure 7 shows differentiation of the HA sample.

 Figure 8 shows differentiation of the HA+CQ sample.

 Figure 9 shows differentiation of the HA+TENDON sample.

 Figure 10 shows the maximum differentiation rate found in the HA+CQ+TENDON sample.

Tenogenesis analysis

**Figure 11:**
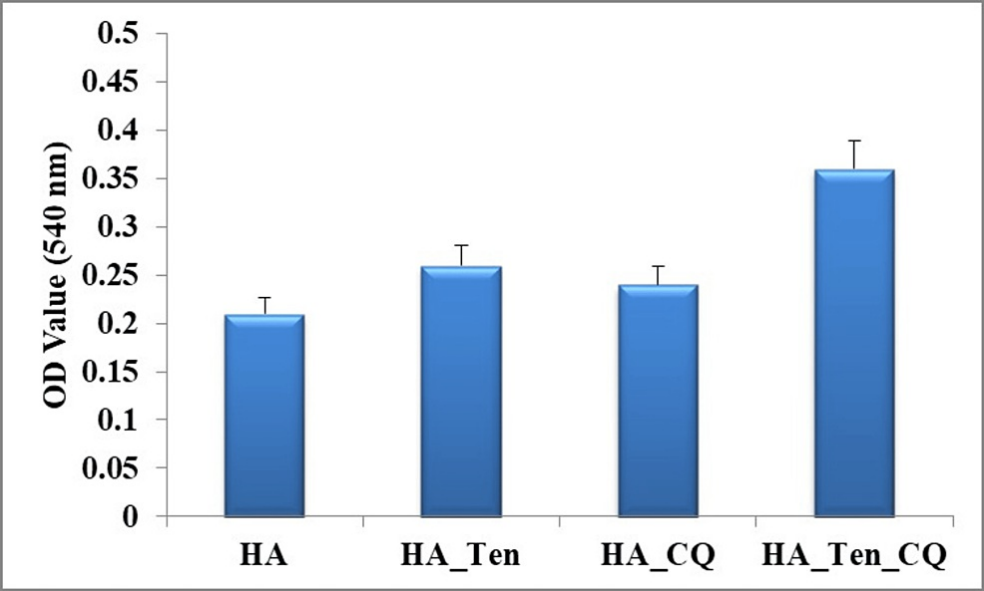
Tenogenesis assay by Picrosirius red staining. The standard deviation in each case is 0.05 (HA), 0.04* (HA_Ten), 0.05 (HA_CQ), and 0.04* (HA_Ten_CQ) * p-value less than 0.05, which is considered statistically significant. HA: hyaluronic acid; HA_Ten: hyaluronic acid + TENDON; HA_CQ: hyaluronic acid +Cissus quadrangularis; HA_Ten_CQ: hyaluronic acid + TENDON +Cissus Quadrangularis.

The tenogenic potential of the stem cells of the corresponding study groups was analyzed by picrosirius red staining, the results of which are shown in Figure 11. From the results, the highest rate of tenogenesis was shown by the stem cell sample present in the HA+TEN+CQ group.

## Discussion

There are many synthetic scaffolds on the market today that are used for regenerative periodontal applications; nevertheless, natural materials are favored over synthetic hydrogels because they offer greater biocompatibility and reduced cytotoxicity. Studies on the application of hydrogel scaffolds for periodontal regeneration are numerous and well-reported [17,18], but there are few on the utilization of natural products and tendon extracellular matrix (ECM) in HA hydrogels. The goal of this work was to identify an appropriate natural substance that could be added to HA hydrogel scaffolds to boost the capacity for periodontal regeneration. This study investigated whether doping CQ extract increases the regenerative potential of mesenchymal stem cells produced in HA scaffolds. CQ was chosen as the natural product for doping in this study because of its use in traditional medicine and its well-known anti-osteoporotic activity. Scanning electron microscopy images were taken to analyze the morphology of the prepared samples. The sample containing HA was observed to have an irregular and rough morphology with cresting and troughing on the topographical region. It was observed that the morphology of the tendon, which has an interconnected fibrous-like appearance, indicated the presence of collagen bundles. When HA and tendon were taken in conjunction, collagen fibers bundled over the HA particles with a regular morphology were observed, and the topographical surface had a highly irregular morphology. The addition of CQ produces a microbead-like appearance due to its interaction with HA. From the above observations, it was inferred that adding a natural product will induce structural changes in the surface morphology of HA. In a previous study, the hydrogel was prepared from decellularized and demineralized bovine bone extracellular matrix, where they found that like collagen type I, hydrogels had a randomly oriented fibrillar structure [19].

Our study is the first of its kind to incorporate a tendon matrix from an ovine source with HA and CQ to make a periodontal regenerative scaffold. Swelling analysis was conducted on the hydrogel samples to analyze the extent of expansion of dimensions due to the absorption of tissue fluid present around its placement area. Minimum to non-existent levels of swelling were considered preferable because, in the event of considerable swelling, it poses a threat to the surrounding tissue and its underlying structures in its region of placement. The swelling ratio percentage has shown a reduction due to doping as observed in HA from 32.5% (approx.) to 20.5% (approx.) in the HA+TENDON+CQ sample. The hydrogel scaffolds were subjected to mechanical compressional analysis to analyze the durability and mechanical stress tolerances. Optimum levels of mechanical strength are required to avoid any form of damage during application in tissues. It was also observed that the maximum compressive strength developed at maximum force during the application fracture was observed in the sample consisting of HA+TENDON (1.38 MPa at 108.72 N), followed by the sample containing HA+CQ+TENDON (0.08 MPa at 6.07 N) then HA+CQ (0.01 MPa at 0.01 N). From these results, it is inferred that the addition of CQ has reduced the mechanical tolerance of the hydrogel scaffolds. The addition of HA has enhanced the mechanical property of the scaffolds, which is in accordance with a previous in vitro study where they used HA and chondrocytes [20]. The cell-secreted ECM is probably a significant component in deciding how strong the hydrogel scaffolds behave in the defect site.

The differentiation analysis was conducted to observe and visualize the proliferation of the stem cells in the HA hydrogel scaffold samples. From the contrast microscopic images taken, similar levels of proliferation of cells in the samples consisting of HA and HA+TENDON+CQ were observed. Also, these samples exhibited greater proliferation levels when compared to the samples consisting of HA+CQ and HA+TENDON. The viability of the hydrogel samples was analyzed using an MTT Compatibility assay to assess the scaffolds' suitability and efficacy when applied in vivo. It was noticed that the highest degree of cell viability was present in the sample containing HA, around 76% (approx.), followed by the sample containing HA+TENDON and HA+CQ, with a viability of around 70% (approx.). The sample consisting of HA+TENDON+CQ obtained a value of approximately 68%. From these results, it is observed that the addition of Cissus quadrangularis with extracellular matrix does not produce a significant reduction of viability in the hydrogel samples.

Biomaterials used in periodontal tissue engineering should be able to replicate the structure and composition of the periodontium, a highly complex tissue, and promote the healing of all periodontal tissues. Alveolar bone regeneration is still very difficult, especially in patients with significant bone resorption and uneven socket damage. New bioactive scaffolds are required for complete periodontal remodeling, which will act as alternatives to existing treatments. Decellularized tendon-derived extracellular matrix (ECM) has been investigated recently as a potential source of complex chemicals that support osteogenic activity and cell proliferation [21,22]. Not much research has been done on the potential of cell-derived ECM along with the herbal component of CQ to promote periodontal regeneration. In our study, Picrosirius red staining was conducted to analyze stem cells' tenogenic activity and proliferation in the hydrogel samples. It was conducted by calculating the absorbance (OD value) at 540 nm. The results obtained showed the maximum proliferation of cells for the sample consisting of HA+TENDON+CQ, with an OD value of 3.5 (approx.), followed by HA+TENDON, with an OD value of 0.25 (approx.). This can be observed as a considerable increase in the sample consisting only of HA, which was observed to have an OD value of 0.2 (approx.). Hence, the addition of Tendon's extracellular matrix and Cissus quadrangularis has increased the tenogenic activity of the HA hydrogel scaffolds. Previous studies have reported that incorporation of bovine tendon extracellular matrix has increased the tenogenic potential of human adipose stem cells [23]. Also, improving the mechanical properties of the hydrogels, apart from increasing the levels of proliferation of stem cells, has proven advantageous [24]. It is possible to create scaffolds with greater bioactivity and complexity that are more similar to the native tissue microenvironment by using cell-derived ECM [25].

Our natural product of choice was the traditional CQ medicinal plant, and the outcomes were encouraging. Plant extracts can be used as essential components of scaffold materials and can also be used as stimulating factors to improve cell adhesion, proliferation, and differentiation [26]. In our study, the CQ+HA+ECM scaffold group showed promising results, where the tenogenic activity of the HA hydrogel scaffold increased with the addition of the CQ plant extract. In a similar study, CQ was added to an alginate and chitosan-based scaffold, and it was found that the herbal CQ scaffold had osteoinductive potential even in the absence of an osteogenic media supplement [27]. The effectiveness of a composite graft made of CQ and bovine hydroxyapatite was assessed in a pilot clinical study. An intrabony defect site was treated with the composite graft in the test group and with only bovine hydroxyapatite in the control group. Despite the fact that both groups had positive benefits, the experimental group's performance was marginally superior [28]. In a previous experiment, a hydroxyapatite, calcium sulfate, and HA-based composite scaffold was studied in a rat model and found to be effective in alveolar bone regeneration [29].

There are a few limitations to this study, as it is a very preliminary in vitro study to check the regenerative potential of the combination of the natural herbal product CQ with ECM of the tendon in an HA hydrogel. One limitation of our study is that the compressive strength parameter was found to be slightly less in the HA+CQ+TENDON group. Despite being ideal, the mechanical tolerance values achieved in this investigation were not greater than the control samples of the HA+TENDON group. The MTT assay showed the lowest value of cell viability for the HA+CQ+TENDON group sample when compared to the control samples. In the future, the regenerative potential of this scaffold can be analyzed by micro- or nano-computed tomography. It is necessary to investigate whether the active ingredients in CQ have influenced the outcomes of the tests carried out for this study. Novel in vitro and animal experiments should be conducted in the future to enhance the swelling rates, and mechanical strength, and boost the cell survival and biocompatibility of herbal-based periodontal regenerative scaffolds.

## Conclusions

Inferring from the results obtained from the study, it can be concluded that doping HA-based hydrogel scaffold with the natural product CQ has increased the tenogenic potential. When the tenogenesis capacity is increased, it also plays a significant role in orienting the collagen fibers, thereby inducing more tensile strength. The doped hydrogel scaffolds were found to be stable and possess superior mechanical properties compared to pure HA hydrogel scaffolds. Thus, Cissus quadrangularis-doped ECM HA hydrogel scaffolds can be a potential candidate for application in surgical therapy for periodontal regeneration due to its increased capacity for tenogenesis.
